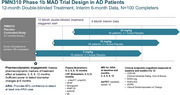# PRECISE‐AD, A Phase 1b, Double‐Blind, Placebo‐Controlled, Multiple Ascending Dose Study of the Safety, Tolerability, Pharmacokinetics, Pharmacodynamics, and Preliminary Efficacy of PMN310 in Patients with Early Alzheimer's Disease

**DOI:** 10.1002/alz70859_103159

**Published:** 2025-12-25

**Authors:** Larry D Altstiel, Misty Lamendola, Wendy Luca, Gavin Malefant, Ebrima Gibbs, Johanne Kaplan, Neil Cashman

**Affiliations:** ^1^ ProMis Neurosciences, Cambridge, MA USA; ^2^ University of British Columbia, Vancouver, BC Canada

## Abstract

**Background:**

Toxic Aβ oligomers (AβO) are implicated in the progression of Alzheimer’s disease (AD). PMN310 is a humanized IgG1 monoclonal antibody that binds to a computationally derived three‐dimensional epitope specific to misfolded Aβ in AβO. Because PMN310 inhibits toxicity of AβO and does not bind to plaque, thereby potentially limiting risk of ARIA, it is being developed as a therapy for early AD. Results from a single ascending dose study of PMN310 (A Phase 1a Study of PMN310 In Healthy Volunteers NCT06105528) indicate that PK parameters and CSF concentrations of PMN310 were linearly dose‐dependent and CSF concentrations were 100‐600 times the estimated AβO molar concentration. The plasma half‐life (t ½) was approximately 17.5 days and the CSF t ½ was approximately 27 days.

**Methods:**

PRECISE‐AD, NCT06750432, is a placebo‐controlled, multiple‐ascending dose study of PMN310 to evaluate safety, tolerability, PK, PD, and preliminary efficacy of multiple intravenous infusions of PMN310 in patients with early Alzheimer's disease. The study consists of three staggered dosing arms of 350 mg, 700 mg, 1400 mg. Patients will be randomized 3:1, PMN310: placebo and will receive either PMN310 or placebo once every 28 days for a total of 12 infusions. The study will enroll 128 patients with either Stage 3 or 4 AD. Diagnosis will be determined by clinical criteria, CSF and plasma biomarkers, and Aβ PET. MRI scans will be done at months 2, 4, 6, 9, 12 to detect potential ARIA. Plasma biomarkers (pTau217, pTau 243, GFAP, SNAP25, neurogranin, Aβ42/Aβ40, NfL) will be measured at baseline and at 3‐month intervals. Biomarkers in CSF will be measured at baseline, 6, 12 months. Cognitive outcomes (CDR‐SB, ADAS‐cog, ADAS‐ADL IADRS Clinical Impression of Change) will be assessed at baseline, month 6 and month 12.

**Results:**

The proposed study has sufficient power to detect at least one ARIA event. The proposed sample size has sufficient power to provide statistically meaningful insight into effects of PMN310 on biomarkers and clinical outcomes.

**Conclusions:**

PRECISE‐AD will be the first study to examine the effects of a monoclonal antibody directed solely against AβO on biomarkers associated with AD pathology and clinical outcomes.